# Basal Mild Dehydration Increase Salivary Cortisol After a Friendly Match in Young Elite Soccer Players

**DOI:** 10.3389/fphys.2018.01347

**Published:** 2018-09-26

**Authors:** Mauricio Castro-Sepulveda, Rodrigo Ramirez-Campillo, Felipe Abad-Colil, Camila Monje, Luis Peñailillo, Jorge Cancino, Hermann Zbinden-Foncea

**Affiliations:** ^1^Exercise Science Laboratory, School of Kinesiology, Faculty of Medicine, Universidad Finis Terrae, Santiago, Chile; ^2^Laboratory of Human Performance, Research Nucleus in Health, Physical Activity and Sport, Department of Physical Activity Sciences, Universidad de Los Lagos, Osorno, Chile; ^3^Facultad de Ciencias de la Educación, Universidad San Sebastián, Valdivia, Chile

**Keywords:** hydration, hormone, endocrine, saliva, catabolic, football, recovery, sport health

## Abstract

A soccer match induce changes in physiological stress biomarkers as testosterone (T), cortisol (C), and testosterone:cortisol (T:C) ration. Hydration state may also modulate these hormones, and therefore may alter the anabolic/catabolic balance in response to soccer match. The role of hydration status before the match in this biomarkers has not yet been reported. The aim of this study was to compare the salivary T, C, and the T:C ratio responses after two friendly matches in well-hydrated and mild-dehydrated (MD) elite young male soccer player. Seventeen players (age, 16.8 ± 0.4 years; VO_2_max 57.2 ± 3.6 ml/kg^−1^/min^−1^) were divided into two teams. Before the matches the athletes were assessed for hydration level by the urine specific gravity method and divided for the analysis into well-hydrated (WH; *n* = 9; USG < 1.010 g/mL^−1^) and mild-dehydrated (MD; *n* = 8; USG 1.010 to 1.020 g/mL^−1^) groups. Hormones were collected before and after each match by saliva samples. The mean (HRmean) and maximal (HRmax) heart rate were measured throughout the matches. A two-way ANOVA was used to compare T, C, and T:C between and within groups. Similar HRmean (WH, 83.1 ± 4.7%; MD, 87.0 ± 4.1; *p* = 0.12) and HRmax (WH, 93.2 ± 4.4%; MD, 94.7 ± 3.7%; *p* = 0.52) were found for both groups during the matches. No differences were found before the matches in the T (*p* = 0.38), C (*p* = 66), nor T:C (*p* = 0.38) between groups. No changes within groups were found after matches in neither group for T (WH, *p* = 0.20; MD, *p* = 0.36), and T:C (WH, *p* = 0.94; MD, *p* = 0.63). Regarding the C, only the MD group showed increases (28%) after the matches (MD, *p* = 0.03; WH, *p* = 0.13). In conclusion MD group exacerbate the C response to friendly matches in elite young male soccer players, suggesting that dehydration before match may be an added stress to be considered.

## Introduction

Hormonal response to a soccer match is a hot topic, with anabolic (i.e., testosterone [T]) and catabolic (i.e., cortisol [C]) hormones potentially influencing the performance and health status of the athlete (Slimani et al., 2017). At the same time, their ratio testosterone:cortisol (T:C) is considered a physiological stress indicator associated to overtraining (Hayes et al., 2015).

Some studies such as Penailillo et al. (2015) showed a decrease on T, without changes on C, whereas others such as Thorpe and Sunderland (2012) describe increases on both hormones. The differences between studies can be explained due to differences in match intensity, biological, psychological, social factors and/or due to the degree or years of training, the latter being highly dependent to the age of the athletes (Casto and Edwards, 2016). In addition, it has been shown that the hormonal response during exercise might depend on hydration state (Roy et al., 2001). For instance, it has been observed greater C concentration on hypohydrated subjects before and after running at 70% of maximal oxygen consumption (VO_2_max) in comparison to euhydrated runners (Maresh et al., 2006). Although in male soccer players, salivary cortisol, T, and T:C have been assessed as physiological stress biomarkers after the match, the role of hydration status before the match in this biomarkers has not yet been reported. This information could increase our understanding of the physical stress induced by a football match, which could improve the preparation and strategy to protect and/or enhance elite performance in subsequent matches. Therefore, the aim of this investigation was to assess the effects of hydration level before a soccer match on the T, C, and T:C response after the match in young elite soccer players.

## Materials and Methods

### Subjects

Seventeen male soccer players (age: 16.8 ± 0.4 years; body mass: 67.5 ± 7.5 kg; height: 173 ± 6.8 cm; VO_2_max: 57.2 ± 3.6 ml/kg^−1^/min^−1^), from a South American under-17 (U17) soccer national team, participated in this study. According to the hydration level assessed by the urine specific gravity (USG) before the matches, the subjects were divided for the analysis into two groups: well-hydration (WH) (USG < 1.010 g/mL^−1^, *n* = 9) and mild dehydration (MD) (USG from 1.010 to 1.020 g/mL^−1^, *n* = 8) (Casa et al., 2000). Injured players and goalkeepers were excluded. The legal guardians of the players signed an informed consent, while the players give their verbal assent, after the potential benefits, and risks where explained to them. The study was approved by the ethics committee of the Universidad Finis Terrae and conformed to the principles outlined in the Declaration of Helsinki.

### Study Design

One week before the matches VO_2_max was assessed to all participants with an incremental test. In the matches day the evaluated soccer team was divided into two teams (A and B). The first match (team A) was played at 11:00 am, and the second match (team B) at 11:30 pm (the same day), in preparation for the FIFA U17 World Championship 2015, carried out in Chile. Climatic conditions were similar between matches. The both friendly matches were played against a professional soccer team of the Chilean professional league. The assessed teams won both matches (first match, 2-0; second match, 3-1). During the two matches, the players were asked to play as it was an official match. The matches follow international rules (FIFA). The USG was assessed 30 min before the match with a portable Refractometer (Robinair, model SPX, United States) in triplicate according to previous suggestions (Castro-Sepulveda et al., 2015). Nutritional recommendations were not made prior to matches and during the friendly matches players consumed water *ad libitum*. According to Penailillo et al. (2015), for the assessment of the T and C, saliva was collected 30 min before each match (Pre-), and 5–10 min after each match (Post-). Briefly, the players were sat, with their eyes open, their head slightly tilted forward and making minimal orofacial movement. All saliva (± 3 ml) was collected for about 2 min. The saliva samples were centrifuged at 1,500 *g* for 15 min and stored at −20°C until analysis. The T and C were determined by enzyme immunoassay using a commercial kit (Salimetrics, State College, PA, United States). The optical density was determined with a microplate reader (Multiskan, Thermo^®^) at 450 nm. All analyses were performed in duplicate according to the manufacturer’s procedures. The intra-assay coefficient of variation was 2.5 and 2.8% for the T and C, respectively. Only players that played >80 min were considered. The mean (HRmean) and maximal (HRmax) heart rate was measured throughout the match using the Polar Team system (MARCA, PAIS). The hearth rate values were reported as relative values, according to age-expected maximum values (220-age).

### Maximal Oxygen Uptake

The VO_2_max was determined by a breath-by-breath pulmonary gas exchange system (Ergocard, Medisoft, Belgium) during an incremental treadmill test. The starting speed was 3 km^−1^/h^−1^, with speed increments of 1 km^−1^/h^−1^ every 60 s. Prior to the tests, the gas analyser was calibrated using gases of known concentrations (VO_2_ = 16.0% and VCO_2_ = 4.0%), and the airflow was calibrated using a 3-liter syringe (Hans Rudolph, Kansas, MO, United States).

### Body Mass Loss

Body mass loss (kg) was calculated by measuring body mass before and after matches (body mass after match – body mass before match) using the same scale (SECA model M20812, Germany), with a precision of 0.1 kg.

### Statistical Analysis

Data is shown as mean ± standard deviation (SD). The normality of the data was analyzed by the Shapiro–Wilk test, showing that data was normally distributed. An unpaired *t*-test was used to compare hydration level, basal characteristics, body mass loss, and HR during matches between groups. A two-way ANOVA was used for the comparison of the T, C, and T:C between and within of WH and MD groups, with a Tukey *post hoc* test when significate main effect was found. The alpha value was set at *p* < 0.05. Statistical analyses were performed in GraphPad Prism^®^ 6.0 (GraphPad Software, San Diego, CA, United States).

## Results

In A team were found five players in WH condition and four players in MD condition and in B team were found four players in WH condition and four players in MD condition. As expected, the USG was lower (*p* < 0.0001) in the WH group (1.006 ± 0.002 g/mL^−1^) compared to the MD group (1.014 ± 0.002 g/mL^−1^). No differences were found between groups in basal characteristics (age: WH 16.3 ± 0.7 and MD 16.9 ± 0.5 years, *p* = 0.61; body mass: WH 68.5 ± 8.6 and MD 67.3 ± 8.4 kg, *p* = 0.68; height: WH 171 ± 9.1 and MD 175 ± 8.0 cm, *p* = 0.21; VO_2_max: WH 56.3 ± 3.8 and MD 57.8 ± 7.2 ml/kg^−1^/min^−1^, *p* = 0.74). Neither were found differences between groups in HRmean (WH 83.1 ± 4.7% and MD 87.0 ± 4.1%; *p* = 0.12) or HRmax (WH 93.2 ± 4.4% and MD 94.7 ± 3.7%; *p* = 0.52) during the matches. After the matches no differences between groups were found in body mass loss (WH 1.6 ± 0.3 kg and MD, 1.4 ± 0.7 kg; *p* = 0.33).

**FIGURE 1:**
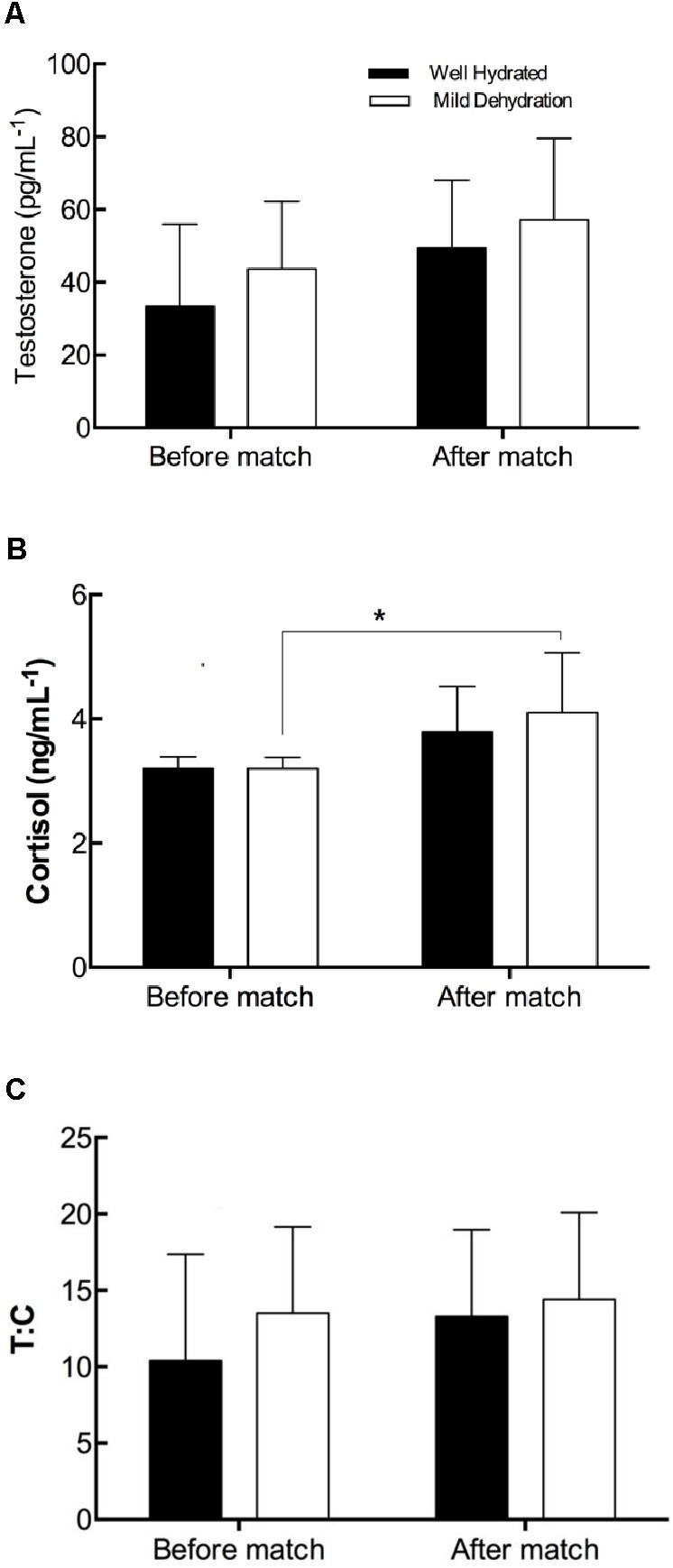
Comparison within well and mild dehydration groups in testosterone **(A)**, cortisol **(B)**, and their quotient **(C)** before and after a friendly match in elite young soccer players. ^∗^*p* < 0.05.

Before the match no differences were found between groups in salivary T (WH 33.41 ± 22.56 pg/mL^−1^ and MD 43.70 ± 18.52 pg/mL^−1^; *p* = 0.38), C (WH 3.17 ± 0.19 pg/mL^−1^ and MD 3.22 ± 0.18 pg/mL^−1^; *p* = 0.66), nor T:C (WH 10.41 ± 6.96 and MD 13.52 ± 5.65; *p* = 0.38). The within-group analysis show that salivary T did not change after matches in WH (49.4 ± 18.6 pg/mL^−1^, *p* = 0.20) nor MD (57.1 ± 22.5 pg/mL^−1^, *p* = 0.36) (**Figure 1A**). With respect to the C level after the match (MD 4.1 ± 0.9 pg/mL^−1^; WH 3.8 ± 0.7 pg/mL^−1^), the MD group exhibit an increase (28%; *p* = 0.03), while no significant change was observed in the WH group (*p* = 0.13) (**Figure 1B**). Regarding the T:C ratio, no changes were found within group in neither group after matches (WH 13.3 ± 5.7, *p* = 0.94; MD 14.4 ± 5.7, *p* = 0.63) (**Figure 1C**). No relationship was found between HR during matches and changes in C (HRmean vs. changes in C, *r* = 0.13, *p* = 0.37; HRmax vs. changes in C, *r* = 0.25, *p* = 0.17).

## Discussion

The aim of this study was to assess the effects of hydration level before a soccer match on the T, C, and T:C after two friendly matches in young elite soccer players. The main results indicate that MD group showed an exacerbated increase in C response after the matches. These results suggest that C response to soccer match is sensitive to hydration state.

With respect to T, different to our results (no changes after match) Penailillo et al. (2015) showed a decrease in T after a friendly match in elite soccer players. It is possible that the different results with the former study, is the difference in years of training experience of athletes. Penailillo et al. (2015) the players had an average of 26 years of age, while in our study, subjects averaged 17 years of age. Therefore, the greater chronological age, hence greater training experience of soccer players in the study of Penailillo et al. (2015), could have induced a reduced hormone response during a competitive match compared to the less experienced U17 soccer players recruited in our study. In addition, several other factors have been identified to play a role in the hormone response to a soccer match (Kobayashi and Miyazaki, 2015; Slimani et al., 2017).

Similar to Penailillo et al. (2015) our results showed no changes in C after match in WH group. However, our results indicate that the MD group showed a significant increase after the match. Considering that no relationship was found between HR during matches and changes in C (*r* = 0.13–0.25, *p* = 0.17–37), and that VO_2_max (i.e., fitness level) was similar in the WH and the MD groups (56.3 and 57.8 ml/kg^−1^/min^−1^, *p* = 0.74), is unlikely that potential differences in physiological stress during match or differences in fitness level explain the increase in C observed in the MD group and the lack of increase in the WH group. Therefore, the hydration level probably played a key significant role. Regarding the T:C, this marker did not change at the end of the match. This may be due to the fact that T:C only decreases during periods of high intensity training, but remains stable during periods of competition (Filaire et al., 2001). In this sense, the role of the hydration level probably played a minor role on the T:C ratio as compared to its role on C levels after the match.

Our study is the first to assess the effects of MD before the match on the response of T, C, and T:C in young elite male soccer players. Our results showed that MD group increase the C response after two friendly matches without alters in effort intensity during matches evaluated by heart rate. Sensibility of C to hydration state was observed previously in seven adults with different levels of hydration completed a strength test. They showed that the most dehydrated subjects (−5% of body weight) showed greater increases in C after exercise, with no changes in T (Judelson et al., 2008). Moreover, another study showed that C increased in hypohydrated (USG = 1,034 ± 0.001 g/mL^−1^) young cross-country athletes (Maresh et al., 2006). Finally, a recent study reported that body weight loss during the match (dehydration) was associated with the increases in C levels in male professional tennis players (López-Samanes et al., 2018). Therefore, our results, in a collective sport, confirm previous findings, expanding the knowledge for young elite male soccer players, regarding the effects of dehydration on stress response (i.e., C increase) during a soccer match.

The C concentrations is a well-recognized physiological stress marker. This steroid hormone plays an important role in response to stress and skeletal muscle recovery after exercise because of the activation of the hypothalamic-pituitary-adrenocortical axis. This finding may be of upmost importance for coaches and medical staff of football teams to consider, since MD before training or competition is very common in football soccer players (Castro-Sepulveda et al., 2015). A previous study in Cushing’s syndrome shows a relationship between HR and C (Chandran et al., 2013). Our results do not show this relationship, this inconsistency could be explained by the different mechanisms that modify the HR in the Cushing’s syndrome and during the exercise.

One of the limitations of this study is not having evaluated other variables that influence C levels and responses as (1) sleep quality before the matches (Bassett et al., 2015) and (2) natural daily response of C (Pritchard et al., 2017). Another potential limitation is that players were free to hydrate during the match. This, although ethically sound, may have altered the after-match hydration level. However, after the match no differences between groups were found in body mass loss (WH, 1.6 ± 0.3; MD, 1.4 ± 0.7; *p* = 0.33).

## Conclusion

In conclusion, MD before soccer match increase C response after match. These results show that C response to soccer match is sensitive to hydration state which suggests that dehydration before match may be an added stress to be considered.

## Author Contributions

MC-S and HZ-F designed the study. MC-S, RR-C, and HZ-F collected and analyzed the data. MC-S, RR-C, FA-C, CM, LP, JC, and HZ-F interpreted the data and prepared the manuscript. All authors approved the final version of the paper.

## Conflict of Interest Statement

The authors declare that the research was conducted in the absence of any commercial or financial relationships that could be construed as a potential conflict of interest.
